# Viral Vector Malaria Vaccines Induce High-Level T Cell and Antibody Responses in West African Children and Infants

**DOI:** 10.1016/j.ymthe.2016.11.003

**Published:** 2017-02-22

**Authors:** Carly M. Bliss, Abdoulie Drammeh, Georgina Bowyer, Guillaume S. Sanou, Ya Jankey Jagne, Oumarou Ouedraogo, Nick J. Edwards, Casimir Tarama, Nicolas Ouedraogo, Mireille Ouedraogo, Jainaba Njie-Jobe, Amidou Diarra, Muhammed O. Afolabi, Alfred B. Tiono, Jean Baptiste Yaro, Uche J. Adetifa, Susanne H. Hodgson, Nicholas A. Anagnostou, Rachel Roberts, Christopher J.A. Duncan, Riccardo Cortese, Nicola K. Viebig, Odile Leroy, Alison M. Lawrie, Katie L. Flanagan, Beate Kampmann, Egeruan B. Imoukhuede, Sodiomon B. Sirima, Kalifa Bojang, Adrian V.S. Hill, Issa Nébié, Katie J. Ewer

**Affiliations:** 1 The Jenner Institute Laboratories, University of Oxford, Old Road Campus Research Building, Oxford OX3 7DQ, UK; 2 Medical Research Council Unit, Fajara, The Gambia; 3 Centre National de Recherche et de Formation sur le Paludisme, Ouagadougou, 01 BP 2208 Ouagadougou, Burkina Faso; 4 Centre for Clinical Vaccinology and Tropical Medicine, The Jenner Institute, Churchill Hospital, Oxford OX3 7LE, UK; 5 Keires AG, Baumleingasse 18, 4051 Basel, Switzerland; 6 European Vaccine Initiative, Universitäts Klinikum Heidelberg, Voßstr. 2, 69115 Heidelberg, Germany; 7 Department of Paediatrics, Imperial College London SW7 2AZ, UK

**Keywords:** malaria, vaccine, antibodies, viral vectors, T cells, Phase I trial

## Abstract

Heterologous prime-boosting with viral vectors encoding the pre-erythrocytic antigen thrombospondin-related adhesion protein fused to a multiple epitope string (ME-TRAP) induces CD8^+^ T cell-mediated immunity to malaria sporozoite challenge in European malaria-naive and Kenyan semi-immune adults. This approach has yet to be evaluated in children and infants. We assessed this vaccine strategy among 138 Gambian and Burkinabe children in four cohorts: 2- to 6-year olds in The Gambia, 5- to 17-month-olds in Burkina Faso, and 5- to 12-month-olds and 10-week-olds in The Gambia. We assessed induction of cellular immunity, taking into account the distinctive hematological status of young infants, and characterized the antibody response to vaccination. T cell responses peaked 7 days after boosting with modified vaccinia virus Ankara (MVA), with highest responses in infants aged 10 weeks at priming. Incorporating lymphocyte count into the calculation of T cell responses facilitated a more physiologically relevant comparison of cellular immunity across different age groups. Both CD8^+^ and CD4^+^ T cells secreted cytokines. Induced antibodies were up to 20-fold higher in all groups compared with Gambian and United Kingdom (UK) adults, with comparable or higher avidity. This immunization regimen elicited strong immune responses, particularly in young infants, supporting future evaluation of efficacy in this key target age group for a malaria vaccine.

## Teaser

An effective malaria vaccine is an urgent global health priority. In these studies, Ewer and colleagues describe strong T cell and antibody responses in children and infants following vaccination with a viral vectored vaccine regime encoding a pre-erythrocytic malaria antigen. This regime has previously demonstrated efficacy in adults and these data support assessment of the efficacy of this vaccine in infants.

## Introduction

Vaccination is one of the most cost-effective health care interventions available, and currently used vaccines prevent an estimated 2.5 million deaths each year.^1^ Most vaccines are administered during infancy and protect primarily through the induction of antibodies.^2^, ^3^ The exception is Bacille Calmette-Guérin (BCG), where protection appears to involve mainly CD4^+^ T cells. However, there is a range of diseases affecting infants, for which vaccines are yet to be developed, where an ability to induce potent CD8^+^ T cell responses could be important. These include r*espiratory syncytial virus* (*RSV*), HIV, tuberculosis, and vaccines targeting malaria parasites at the liver stage of infection.^4^ An extensive literature in murine immunology documents frequent reductions in CD8^+^ T cell induction in newborn mice, suggesting that generation of such T cell responses in human infants might be difficult.^5^, ^6^, ^7^, ^8^ Limited studies in infants demonstrate reduced Th1 and proliferative responses to vaccination;^9^, ^10^, ^11^ however, data are lacking on the capacity to induce CD8^+^ T cells in early infancy.

A highly effective malaria vaccine against the most lethal malaria species, *Plasmodium falciparum*, could help to save half a million lives each year.^12^ The primary target population for a vaccine is young infants in sub-Saharan Africa because from 6 months of age, infants and children in this region bear the greatest burden of malaria mortality.^12^ The most advanced malaria vaccine, RTS,S/AS01, shows good efficacy against controlled human malaria infection (CHMI) in adults in the United States,^13^, ^14^ however, efficacy against clinical malaria observed among 6- to 12-week-old infants in a large phase 3 clinical trial was ∼30% over 12 months,^15^, ^16^ and declined thereafter, well below the target of 75% efficacy against clinical malaria specified by the updated Malaria Vaccine Technology Roadmap.^17^, ^18^ RTS,S/AS01 does not induce CD8^+^ T cells; efficacy is mediated by IgG antibodies and CD4^+^ T cells against the circumsporozoite (CS) protein, a pre-erythrocytic antigen that is highly abundant during the sporozoite stage of the parasite life cycle.^19^, ^20^, ^21^, ^22^ Immunogenicity data from efficacy trials demonstrated that the levels of anti-CS antibodies induced in 6- to 12week-old infants were 3-fold lower than in 5- to 17-month-olds, suggesting that RTS,S/AS01 is less immunogenic in young infants.^15^, ^23^ This stage of the parasite life cycle is an attractive target for a humoral response as sporozoites can be eliminated before infecting host hepatocytes, however, this window may be as brief as 30 min.^24^ The liver-stage of the *P. falciparum* life cycle is also a leading target for vaccination. This stage lasts between 5.5 and 7 days in humans,^25^, ^26^, ^27^ thus prolonging the opportunity for antigen-specific CD8^+^ T cells to locate and kill infected hepatocytes.

We have previously described vaccination approaches employing the sporozoite antigen thrombospondin-related adhesion protein (TRAP) fused to a multiple epitope string (ME) in a number of delivery platforms including DNA and replication-deficient viral vectors.^28^ Most recently, we have demonstrated the safety and immunogenicity of a heterologous prime-boost approach using a chimpanzee adenovirus (ChAd63) and modified vaccinia virus Ankara (MVA), both encoding the ME-TRAP subunit.^29^, ^30^, ^31^ This regimen induces cellular immunity comprising both CD4^+^ and CD8^+^ phenotypes and IgG antibody responses in malaria-naive and semi-immune adults.^32^, ^33^ Against CHMI with *P. falciparum*-infected mosquitoes, ChAd63 MVA ME-TRAP elicited 21% sterile efficacy and significantly delayed the time-to-patency of malaria in a further 36% of vaccinees.^34^ Efficacy was strongly associated with monofunctional interferon-gamma (IFNγ)-secreting CD8^+^ T cells. In a recent field trial in Kenyan adults, 67% efficacy against malaria infection was induced by the same immunization regime, and again, a T cell correlate of efficacy was observed.^35^ Significant anti-TRAP IgG titers after heterologous prime-boost with ChAd63 ME-TRAP and MVA ME-TRAP could also contribute to vaccine efficacy.^33^

We present here a detailed evaluation of the high-level T cell and antibody responses induced by this regimen in young children and infants. Infants have very different hematological parameters to older children and adults: total blood volume is substantially lower and numbers of circulating lymphocytes per ml significantly higher.^36^ We propose an alternative approach for calculating T cell responses following vaccination, which takes account of the higher lymphocyte count in infants and young children. This methodology is especially relevant when comparing cellular immunogenicity across age groups.

Here, we report T cell and antibody immunogenicity across four pediatric age strata ranging from 6 years to 10 weeks old, from three clinical vaccine studies using the ChAd63 ME-TRAP and MVA ME-TRAP regimen in malaria-exposed African children and infants. Responses are also compared to those of malaria-naive and malaria-exposed vaccinated adults. Given that each clinical trial was a small phase I study, combining the datasets together into a single analysis facilitates the observation of trends across age groups and between cohorts with differing malaria exposure. Two clinical trials were performed in the western region of The Gambia in Sukuta, where modest malaria transmission is still observed following seasonal rains, despite a substantial decline in incidence since 2003.^37^ A third study was undertaken in Burkina Faso with highly seasonal transmission and a far greater incidence of malaria than in The Gambia, averaging more than one confirmed clinical episode per child per year.^38^, ^39^ An effective malaria vaccine would be useful in both settings.

## Results

### Study Design

Two hundred children were screened for eligibility across the three trials and 138 eligible children were enrolled, vaccinated, and followed up (Figures S1A and S1B) Trial groups are shown in Table 1. Primary outcomes of safety, reactogenicity, dose-finding, and preliminary cellular immunogenicity from this study are reported separately.^40^ Baseline demographics of trial participants are shown in Table S1.

**Table 1:** Study Outline

Group	Age	Site	N	1^st^ Vaccine Dose ChAd63 ME-TRAP, vp	2^nd^ Vaccine Dose MVA ME-TRAP, PFU
1a	2–6 years	The Gambia	6	1 × 10^10^	1 × 10^8^
1b			6	1 × 10^10^	2 × 10^8^
1c			6	HDCRV (1 mL)	HDCRV (1 mL)
1d			6	5 × 10^10^	1 × 10^8^
1e			6	5 × 10^10^	2 × 10^8^
1f			6	HDCRV (1 mL)	HDCRV (1 mL)
2a	5–12 months	The Gambia	12	1 × 10^10^	1 × 10^8^
2b			12	5 × 10^10^	1 × 10^8^
2c			12	no vaccine	no vaccine
3a	10 week	The Gambia	12	1 × 10^10^	1 × 10^8^
3b			12	5 × 10^10^	1 × 10^8^
3c			12	no vaccine	no vaccine
4	5–17 months	Burkina Faso	30	5 × 10^10^	1 × 10^8^

vp, viral particles; PFU, plaque-forming units; HDCRV, human diploid cell rabies vaccine.

### Immunogenicity

#### T Cell Responses Assessed by ELISpot

**Figure 1:**
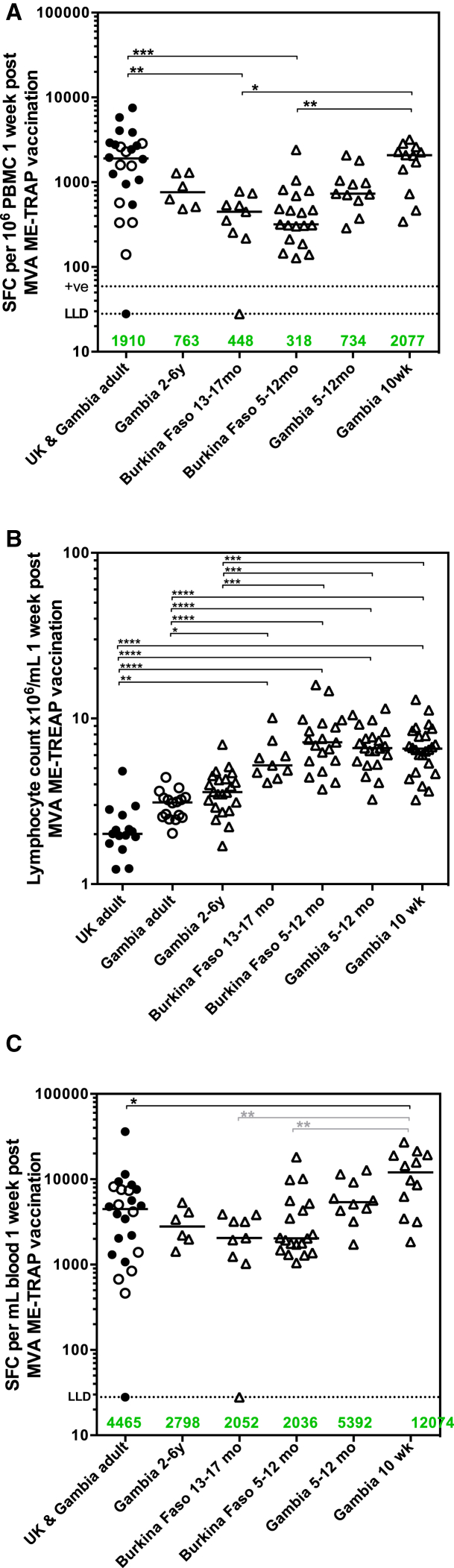
ELISpot Responses across Trials with Age De-escalation (A) Comparison of peak ELISpot immunogenicity at 7 days post-MVA including data from previously published trials in adults,^32^, ^34^ expressed as SFC per 10^6^ PBMC (Kruskal-Wallis test with Dunn’s post-test for multiple comparisons between all groups. Dotted lines show lower limit of assay detection (LLD) and positive threshold for ELISpot response. (B) Lymphocyte counts per milliliter of blood for all vaccinated volunteers. (Kruskal-Wallis test with Dunn’s post-test for multiple comparisons between all groups. Adult groups not combined due to difference by 2-tailed Mann-Whitney test, p = 0.0009). (C) ELISpot responses expressed as SFC per milliliter of blood (Kruskal-Wallis test with Dunn’s post-test for multiple comparisons to adult control group denoted by black bars, Kruskal-Wallis test with Dunn’s post-test for multiple comparisons between all pediatric groups denoted by gray bars). Closed circles, UK adults; open circles, Gambian adults; open triangles, all pediatric groups. Numbers in green and group bars represent group medians. *p < 0.05, **p < 0.01, ***p < 0.001, ****p < 0.0001.

We report elsewhere ex vivo IFNγ ELISpot responses stratified by age and dose in group 1 and group median response data stratified by dose in groups 2, 3, and 4.^40^ Peak ELISpot responses were compared between groups in these age de-escalation studies with those from previously published adult phase I trials in the United Kingdom^34^ and The Gambia.^32^ A trend toward lower T cell immunogenicity in pediatric vaccinees compared with adult vaccinees was measured, with responses in Burkinabe children significantly reduced (Figure 1A). Notably, responses in 10-week-old Gambian infants were comparable to those in adults and higher than in Burkinabe children aged 5–12 months and 13–17 months (Figure 1A; p < 0.0001, Kruskal-Wallis test with Dunn’s correction for multiple tests). No differences were measured between responses in United Kingdom (UK) and Gambian adults (data not shown^32^). Due to the observation that children under 17 months have significantly higher lymphocyte counts per milliliter of blood than older children and adults (Figure 1B; p < 0.0001, Kruskal-Wallis test with Dunn’s correction for multiple tests), expressing ELISpot responses using the number of peripheral blood mononuclear cells (PBMC) as the denominator may not accurately reflect immunogenicity across age groups. We therefore express ELISpot responses as spot-forming cells (SFC) per milliliter of blood by integrating lymphocyte counts collected during routine hematology tests at each time point. Using this arguably more physiologically relevant denominator, T cell responses were comparable between adults, children, and older infants. Furthermore, responses in 10-week-old infants were almost 3-fold higher than UK and Gambian adults (Figure 1C, p = 0.0017, Kruskal-Wallis test with Dunn’s correction for multiple tests against adult control group). No differences were measured between responses in UK and Gambian adults (data not shown). Responses remained higher in Gambian 10-week-old infants compared to both Burkinabe age groups (Figure 1C; p = 0.0007, Kruskal-Wallis test with Dunn’s correction for multiple tests between all pediatric groups).

#### T Cell Responses Assessed by Flow Cytometry

**Figure 2:**
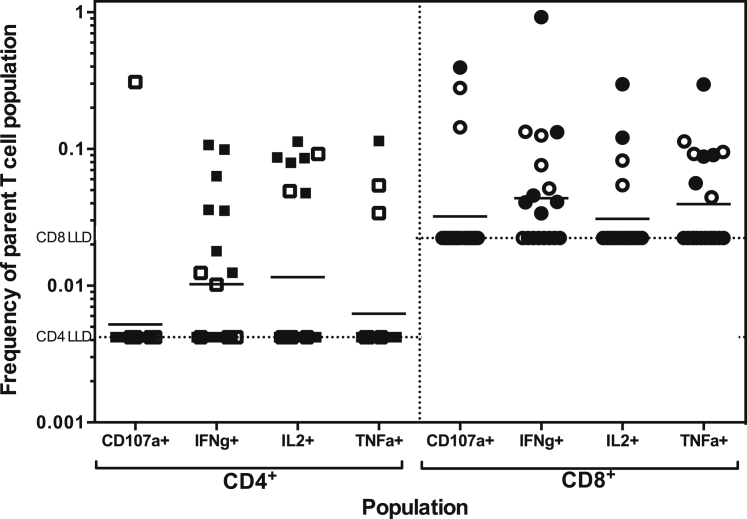
T Cell Responses by Flow Cytometry Responses were assessed from cryopreserved PBMC for group 2 (5- to 12-month-olds in the Gambia, N = 8) and group 4 (5- to 17-month-olds in Burkina Faso, N = 12). Dotted lines represent the lower limit of detection for CD4^+^ and CD8^+^ T cell populations. Bars represent geometric means. Open symbols, group 2; closed symbols, group 4.

Flow cytometry is reported for samples from Gambian 5- to 12-month-olds and Burkinabe 5- to 17-month-olds. Due to limited PBMC availability and technical issues with the assay, older children and young infants were not assessed. Assessment of cytokine expression 7 days after boosting with MVA ME-TRAP showed detectable IFNγ, interleukin 2 (IL-2), and tumor necrosis factor-alpha (TNF-α) secretion from CD4^+^ and CD8^+^ T cells (Figure 2). Expression of the degranulation marker CD107a on CD8^+^ T cells was also evident in 15% of volunteers tested.

#### Antibody Responses after Vaccination

**Figure 3:**
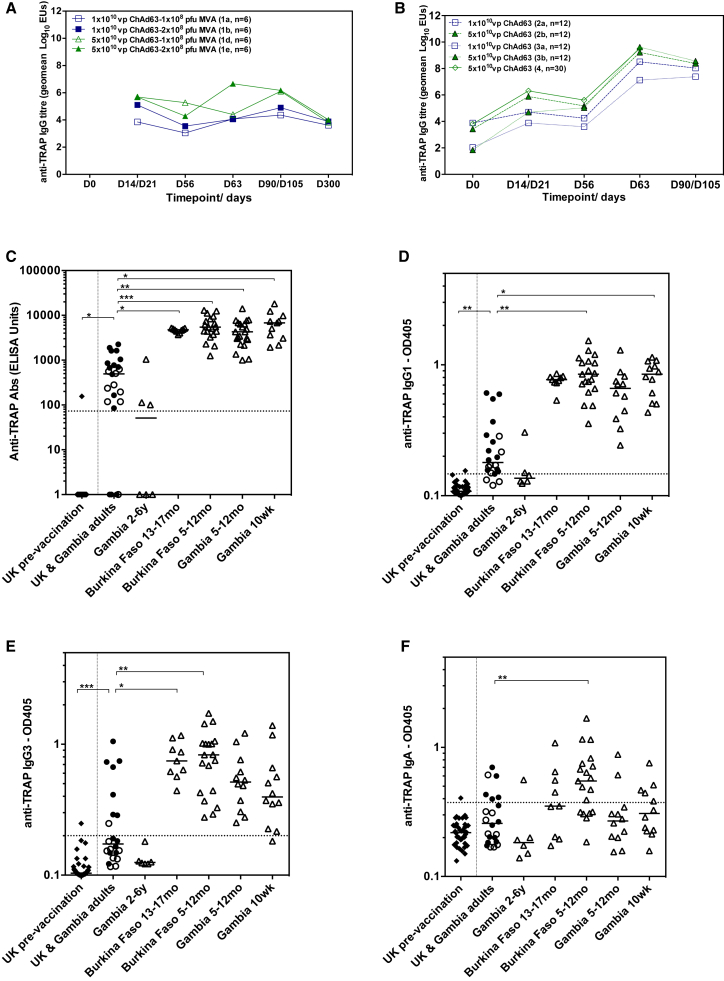
Antibody Responses to Vaccination (A) Geometric mean time course of anti-TRAP IgG for Gambian 2- to 6-year-olds (group 1) vaccinated with high and low dose ChAd63 ME-TRAP and MVA ME-TRAP. (B) Geometric mean time course of anti-TRAP IgG titer for Gambian 5- to 12-month-olds (group 2), Gambian 10-week-olds (group 3) and Burkinabe 5- to 17-month-olds (group 4). (C) Peak IgG titer at 7 days post-MVA. (D and E) TRAP-specific IgG1 and IgG3 antibodies at day 63 (1 week post-MVA ME-TRAP). (F) TRAP-specific IgA antibodies at day 63. Dashed lines show seropositive threshold (mean + 3 SD of 42 malaria-naive samples tested on each assay). Closed circles UK adults at peak; open circles Gambian adults at peak; open triangles all pediatric groups at peak. All adults boosted with 2 × 10^8^ PFU MVA ME-TRAP, all children and infants boosted with 1 × 10^8^ PFU MVA ME-TRAP. Medians displayed. All comparisons across groups made using Kruskal-Wallis with Dunn’s post-test for multiple comparisons to adult control group. *p < 0.05. **p < 0.01, ***p < 0.001, ****p < 0.0001.

Antibody responses to vaccination in Gambian 2- to 6-year-olds (group 1) were weak, irrespective of priming or boosting dose (Figure 3A). IgG titers in Gambian and Burkinabe infants and young children (groups 2, 3, and 4) markedly increased 7 days after boosting with MVA (Figure 3B). In Gambian 10-week-olds (group 3), IgG titers were significantly higher post-boost in the group that received the higher dose of ChAd63 ME-TRAP (p = 0.0008, 2-tailed Mann Whitney test, group 3a versus 3b), however, there were no significant effects of ChAd63 ME-TRAP dose in other age groups (Figure 3B). Responses in all groups of infants and children under 2 years were very substantially and significantly higher than responses in UK and Gambian adults (Figure 3C; p = < 0.0001, Kruskal-Wallis test with Dunn’s correction for multiple tests comparing between all groups). Responses in Gambian 2- to 6-year-olds (group 1) were similar in magnitude to those in adults. IgG subtypes were also measured for each group. IgG1 and IgG3 subclasses showed the highest increase above baseline, with significantly higher titers in infants and young children (groups 2, 3, and 4) than older children (group 1) or adults (Figures 3D and 3E). Titers of IgA antibodies at the peak of the immune response were more frequently detected in the Burkinabe infants, than in the Gambian groups (Figure 3F). IgM antibody responses were low after vaccination and did not vary between groups (data not shown).

**Figure 4:**
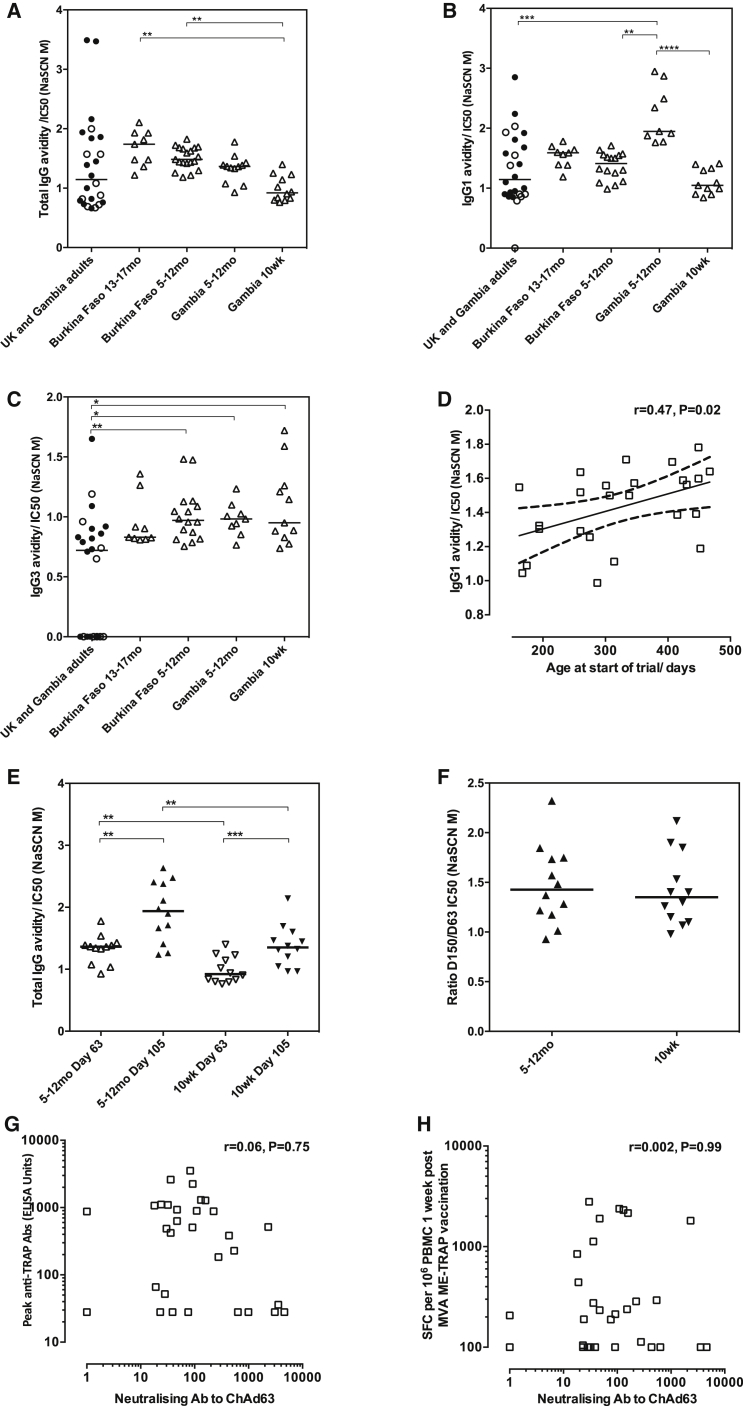
Antibody Avidity and Anti-Vector Neutralizing Antibodies (A) Avidity of total IgG (B), IgG1 and (C) IgG3 subtypes in Burkinabe and Gambian younger children and infants (groups 2, 3, and 4) and adults (Kruskal-Wallis test with Dunn’s post-test for multiple comparisons between all groups). (D) Effect of age at first vaccination on IgG1 antibody avidity after boosting in Burkinabe 5- to 17-month-olds (group 4, Spearman’s r = 0.47, p 0.02). (E) Increase in total IgG avidity between 1 and 7 weeks post boost, (p = 0.0015 for 5- to 12-month-olds, p = 0.0010 for 10-week-olds, Wilcoxon matched pairs for comparisons within groups. Mann Whitney test with post-test for multiple comparisons between groups at comparable time points). (F) Change in total IgG avidity between 1 and 7 weeks post boost, expressed as a ratio for each age group (no significant difference by t test). (G and H) Correlations between group 1 neutralizing antibody titers to the ChAd63 vector and peak antibody titers by ELISA and T cell responses by ELISpot, respectively. Spearman’s r = 0.06, p 0.75 for (G) and r = 0.002, p 0.99 for (H). Medians displayed. Kruskal-Wallis tests performed with Dunn’s post-test for multiple comparisons between all groups. *p < 0.05. **p < 0.01, ***p < 0.001, ****p < 0.0001.

Avidity of IgG antibodies was measured at the peak time point (Figure 4A) and was significantly higher in Burkinabe infants and children when compared with Gambian 10-week-olds (p = 0.0006, Kruskal-Wallis test with Dunn’s correction for multiple tests). A similar trend in IgG avidity was seen in Gambian 5- to 12-month-olds compared to Gambian 10-week-olds. Children aged 2–6 years (group 1) were not included in this analysis due to the low number of individuals with high enough titers to perform the assay. Differences in antibody avidity between groups were further studied by measuring the avidity of IgG1 and IgG3 subtypes in younger children and infants (groups 2, 3, and 4; Figures 4B and 4C), which revealed that the lower IgG avidity in 10-week-olds was largely due to lower avidity of IgG1 antibodies as IgG3 avidity was comparably low across all groups of infants and children. Avidity of IgG1 antibodies was highest in Gambian infants aged 5–12 months and significantly higher than that in Burkinabe infants of comparable age (Figure 4B; p < 0.0001, Kruskal-Wallis test with Dunn’s correction for multiple tests). A clear effect of age was apparent in Burkinabe 5- to 17-month-olds (group 4), in which IgG1 avidity was positively associated with age at first vaccination (Spearman’s r = 0.47, p 0.02, Figure 4D). Total IgG avidity significantly increased between 1 and 7 weeks post-boost in both Gambian 5- to 12-month-olds and 10-week-olds (p = 0.0015 and p = 0.0010, respectively, Wilcoxon matched pairs test between time points within the same group, Figure 4E). The magnitude of the increase in avidity after boosting was the same for both age groups with the ratio of the avidity at 7 weeks to 1 week post-boost comparable for 5- to 12-month-olds and 10-week-olds (Figure 4F).

Neutralizing antibodies to the adenovirus vector were measured prior to vaccination in group 1 only and were weaker than those measured previously in Gambian adults (74 ELISA units [EUs], 95% confidence interval [CI] 35–155 in group 1 versus 192 EUs, 95% CI 104–422).^32^ Neutralizing antibody titers were not correlated with T cell or antibody responses at the peak time point 7 days after MVA ME-TRAP vaccination (Figures 4G and 4H).

## Discussion

We demonstrate here that immunization of children and infants with a prime-boost regimen using ChAd63 and MVA ME-TRAP induces high level T cell and antibody responses to a pre-erythrocytic malaria antigen that has previously demonstrated efficacy against CHMI in malaria naive adults and against natural infection in semi-immune adults.^33^, ^34^, ^35^ Levels of TRAP-specific T cells and IgG were highest in infants who received their priming immunization at 10 weeks of age and an MVA ME-TRAP boost 8 weeks later.

Higher ELISpot responses were measured in adults vaccinees compared to pediatric vaccinees using the standard ELISpot readout. However, comparisons of immunogenicity in de-escalating age groups are complex due to differences in body mass and total blood volume of participants receiving similar doses of vaccine. More specifically, variations are observed in the numbers of lymphocytes circulating per milliliter of blood between adults and children and between infants and older children. To address this, we have incorporated the lymphocyte count from the full blood count performed at the same time into the denominator of the units used to report ELISpot responses. Using this approach to take account of the much higher lymphocyte frequencies measured in young children and infants, we have quantified comparable T cell responses between adults, children, and older infants. Responses in 10-week-old infants were almost 3-fold higher compared with adults, indicating that ChAd63 and MVA ME-TRAP T cell immunogenicity is increased and not reduced with administration to young infants.

While T cell responses between the two sites were comparable between 5- to 17month-olds, overall lower responses were measured in Burkina Faso, potentially due to a combined effect of age and differing malaria exposure by site. Malaria-related immunosuppression could lead to reduced vaccine immunogenicity or differences in innate immunity may exist in the early weeks of life in these children. Congruently, malaria transmission in the Gambia is markedly lower than in Burkina Faso,^37^, ^39^, ^41^ thus these effects would likely be seen in older Gambian children who have had more years of lower level malaria exposure; and in younger infants in Burkina Faso where transmission is higher.^42^, ^43^

Higher frequencies of antigen-specific cells per milliliter of blood trafficking through the spleen may increase the likelihood of antigen recognition from antigen-presenting cells following vaccination, giving rise to greater cellular immunity. The level of antigen-specific T cells per milliliter is likely to correlate better with vaccine efficacy than T cells per million PBMC, as the former should correlate better with the rate at which antigen-specific T cells enter the liver, which is the relevant target organ in malaria.

Anti-TRAP IgG titers at the peak time point were, remarkably, ∼20-fold higher in the youngest groups of children and infants than those at the same time point post-vaccination in malaria-naive and semi-immune adults. Interestingly, titers in 2- to 6-year-old children were lower than those in adults and younger children or infants, which when assessed in combination with the similar observation in T cell frequencies, suggests vaccination of older children may be less effective at preventing malaria infection. A recent study with these viral vectors expressing TRAP and circumsporozoite protein (CS) in malaria-naive adults, proposed a role for both anti-CS and anti-TRAP antibody responses in reducing parasite density during the liver-stage of malaria infection.^33^ This suggests that the additional component of antibody immunogenicity elicited in the youngest age groups here may improve the efficacy of this vaccine regime to a level above that observed in adults undergoing CHMI. In RTS,S/AS01 trials a reduction of anti-CS IgG responses in a comparable group of 6- to 12-week-old infants compared with adults associated with decreased vaccine efficacy.^44^ The finding that viral-vector-induced antibody levels are not decreased in this age group is therefore very encouraging, because this is the preferred target age group for a malaria vaccine.

Avidity of antibodies measured 7 days after boosting was lower in young infants than in 5- to 12- or 5- to 17-month-olds, although the kinetics of avidity maturation were comparable between these age groups. In the Burkinabe infants, IgG1 avidity was significantly lower than in Gambian infants of the same age, perhaps again due to increased malaria exposure in Burkina Faso. Avidity of IgG antibodies alone has been shown not to be predictive of malaria vaccine efficacy for RTS,S/AS01, however, an association between the change in avidity, as well as IgG titers following the second and third dose were strongly associated with a reduction in the risk of malaria.^45^ This suggests that the kinetics of antibody avidity maturation as well as the magnitude of the IgG response contributes to vaccine efficacy. Further work to determine the potential for antibodies induced by this regime to inhibit parasite invasion using in vitro functional assays is underway.

Previous studies have demonstrated a reduction of cellular responses to vaccines in infants with reduced or defective secretion of Th1 cytokines in response to oral polio and protein-in-adjuvant vaccines, such as DTaP.^10^, ^11^, ^46^ Similarly, preferential differentiation of B cells into memory cells rather than plasma cells is associated with reduced IgG responses to protein and polysaccharide antigens in infants.^8^, ^47^ The preliminary observations reported here demonstrate that replication-deficient viral vectors can elicit immune responses in infants that appear to be superior to those in comparable adult populations. Delivery of antigens by viral vectors may overcome the limitations of the immature infant immune system, which have been shown to limit seroconversion to EPI vaccines.^48^, ^49^ This is consistent with strong CTL function previously measured following congenital infection with cytomegalovirus and *Trypanosoma cruzi*, demonstrating that under certain conditions, the immature human immune system is indeed capable of potent CTL activity.^50^, ^51^

In this study, we have assessed the ability of a virally vectored prime boost regime to induce immunity to a protective pre-erythrocytic malaria antigen in two populations with very different malaria endemicities. Furthermore, a novel methodology for more physiological and robust comparison between adult and pediatric T cell responses was developed and applied across several age de-escalating clinical studies. Our immunogenicity findings combined with the acceptable safety profile observed in these studies^40^ show clear potential utility of this approach for immunization against malaria and other childhood illnesses where either antibodies or cellular immunity are relevant to protection, for example RSV.^52^ Further studies are underway to assess optimal regimes for immunization with co-administration of WHO Expanded Program of Immunisation (EPI) vaccines and to determine efficacy against clinical and severe malaria in a cohort of infants and children in a region of high malaria transmission.

## Materials and Methods

### Objectives

The primary objective of these trials was to evaluate the safety and reactogenicity of the ChAd63 ME-TRAP and MVA ME-TRAP vaccines in malaria-experienced Gambian and Burkinabe children. Secondary objectives were to evaluate the cellular and humoral immunogenicity of the vaccines in two settings of varying seasonal malaria transmission while the tertiary objective was to compare the immunogenicity of the low and high doses of MVA ME-TRAP (Gambian children only).

### Study Setting

The first clinical trial (group 1) took place from December 2010 to December 2011 in the Sukuta field site of Medical Research Council, The Gambia. Sukuta is a peri-urban Gambian village located about 30 km south of the capital Banjul. The Sukuta field site previously served as the base for the phase I trials of ChAd63 MVA ME-TRAP vaccine regimen in adults (18). In this region, malaria transmission is highly seasonal, occurring almost exclusively during the rainy season (July to December) with greatest incidence from September to November. *Anopheles gambiae* is the principal malaria vector. Previous studies have documented decline in incidence of malaria in The Gambia.^37^, ^41^ The second clinical trial (groups 2 and 3) took place in the same setting between September 2011 and March 2013.

The third clinical trial (group 4) took place from December 2012 to September 2013 in Banfora Health District in the Cascades region of South Western Burkina Faso, about 400 km southwest from the capital Ouagadougou. Malaria transmission is stable during the year, with increased levels during the rainy season from May to November, peaking from May to September.^38^ *A. gambiae* is the principal malaria vector. Immunogenicity analyses of group 4 are split into two age groups: 5–12 months and 13–17 months, permitting direct analysis of 5- to 12-month-olds in the Gambia and Burkina Faso.

### Ethics and Regulatory Approval

An independent Data Safety and Monitoring Board (DSMB) was appointed before the trials began to provide oversight and to review the safety data reports as the trials progressed. Experienced local pediatricians served as local safety monitors (LSM) and, along with the DSMB, reviewed all safety data between dose escalations. In addition, trials were conducted according to ICH Good Clinical Practice guidelines and were monitored by an external organization (Appledown Clinical Research). The Gambian Government/Medical Research Council Joint Ethics Committee, The Gambia Medicines Board, the Burkina Faso Ministry of Health and Institutional Bioethics Committee, the UK Medicines and Healthcare products Regulatory Authority, and Oxford Tropical Research Ethics Committee (OXTREC Numbers: 64-09, 26-11, 41-12) granted approval of the study protocol. All three trials were registered with https://clinicaltrials.gov (NCT01373879, NCT01450293, NCT01635647) and the Pan African Clinical Trials Registry (www.pactr.org) (PACTR201204000362870, PACTR201401000363170, PACTR201208000404131).

### Study Design

We conducted three phase Ib studies. The first study (group 1, aged 2–6 years) in The Gambia was the pediatric arm of a phase Ib single-blind, randomized controlled, dose-escalation study in adults that has been reported previously.^31^, ^32^ The second study, also in The Gambia, was a subsequent single-blind randomized controlled, dose-escalation study in children aged 5–12 months (group 2) and 10 weeks (group 3) at vaccination with ChAd63 ME-TRAP. The third study was a phase I open-label safety lead-in group of a larger phase IIb study in Burkina Faso in children aged 5–17 months at first vaccination (group 4). CONSORT diagrams are provided in the Supplemental Information. Protocol S1 (group 1, The Gambia), protocol S2 (groups 2 and 3, The Gambia), protocol S3 (group 4, Burkina Faso), and checklists S1–S3 are given in the supplemental information of the paper reporting the clinical outcomes of these studies.^40^ All vaccinations were intramuscular with group 1 receiving doses in the deltoid region of the arm, while all other groups were vaccinated in the anterolateral thigh. A control group was added to group 1 because of the anticipated high frequency of concurrent diseases in the study age group of 2–6 years and also to aid objective assessment of the relationship of adverse events to vaccination. Human diploid cell rabies vaccine (HDCRV) (Sanofi Pasteur MSD) was chosen as the comparator vaccine because rabies is endemic in The Gambia and anti-rabies vaccines were not readily accessible for pre-exposure prophylaxis; hence, the investigators deemed that giving HDCRV might benefit the study participants. In groups 2 and 3, no-treatment controls were included, but there was no control group in group 4 as the subsequent larger phase II study had a rabies vaccine control arm. For group 1, 36 eligible children were randomized to receive either group 1a: low dose ChAd63 ME-TRAP (1 × 10^10^vp) followed by low dose MVA ME-TRAP (1 × 10^8^ plaque-forming units [ PFU]); group 1b: low dose ChAd63 ME-TRAP (1 × 10^10^vp) followed by high dose MVA ME-TRAP (2 × 10^8^ PFU); group 1c: control HDCRV 1 mL followed by HDCRV 1 mL; group 1d: high dose ChAd63 ME-TRAP (5 × 10^10^vp) followed by low dose MVA ME-TRAP (1 × 10^8^ PFU); group 1e: high dose ChAd63 ME-TRAP (5 × 10^10^vp) followed by high dose MVA ME-TRAP (2 × 10^8^ PFU); group 1f: Control HDCRV 1 mL followed by HDCRV 1 mL intramuscular (IM). All vaccinations were separated by an 8-week interval.

For groups 2 and 3, 36 eligible children in each group were randomized to receive either group a: low dose ChAd63 ME-TRAP (1 × 10^10^vp) followed by low dose MVA ME-TRAP (1 × 10^8^ PFU); group b: high dose ChAd63 ME-TRAP (5 × 10^10^vp) followed by low dose MVA ME-TRAP (1 × 10^8^ PFU); group c: no vaccine. For group 4, all children received high dose ChAd63 ME-TRAP (5 × 10^10^vp) followed by low dose MVA ME-TRAP (1 × 10^8^ PFU).

Pediatric groups are compared to malaria naive adult vaccinees in the UK and semi-immune adult vaccinees the Gambia, who all received 5 × 10^10^ viral particles (vp) ChAd63 ME-TRAP and 2 × 10^8^ PFU MVA ME-TRAP.

### Randomization in Groups 1, 2, and 3 and Blinding

An independent statistician at the Centre for Statistics in Medicine, Oxford performed a stratified randomization of participants (stratified by age into two categories and split by the median values of ages of children recruited). The list of eligible children after screening was sent to the statistician who carried out the randomization. The statistician had no knowledge of the participants, except the age, as this was required for the stratification. The children were randomly allocated to six groups in dose-escalated fashion. This was done to determine the tolerable doses as this was the first time ChAd63 and MVA ME-TRAP vaccines were being administered in Gambian children. The investigators and the vaccinators were un-blinded to the group allocations. However, the study children’s parents/carers and field workers who conducted post-vaccination assessment of reactogenicity and solicited symptoms were blinded to the group allocations.

### Sample Size

These phase Ib trials were not powered to detect differences between groups. The sample size was based on general acceptance of this size for initial assessment of safety, tolerability, and immunogenicity of the investigational vaccines in a malaria endemic area and this size balances the need to avoid exposing a large group of study participants to an unknown risk with the need for useful safety and immunogenicity data from an adequate sample size.

### Interventions

The Clinical Biomanufacturing Facility (CBF; University of Oxford, UK) and IDT (Germany) manufactured ChAd63 ME-TRAP and MVA ME-TRAP respectively under Good Manufacturing Practice conditions, respectively as previously described.^34^

### Blood Processing

Blood samples were stored at room temperature prior to processing, which was completed within 6 hr of venepuncture. PBMC were separated by density centrifugation from heparinized whole blood and resuspended in RPMI containing 10% heat-inactivated, batch-tested, sterile-filtered fetal bovine serum (FBS) previously screened for low reactivity (Labtech International), 1% L-glutamine, and 1% penicillin/streptomycin. Cell counts were performed using trypan blue staining and a microscope according to an established standard operating procedure (SOP) in the lab.

### Ex Vivo IFNγ ELISpot Assays

Ex vivo (18 hr stimulation) ELISpot assays were performed using Multiscreen IP ELISpot plates (Millipore), human IFNγ SA-ALP antibody kits (Mabtech), and BCIP NBT-plus chromogenic substrate (Moss Inc.). Cells were cultured in RPMI (Sigma) containing 10% heat-inactivated, sterile-filtered fetal calf serum, previously screened for low reactivity (Labtech International) supplemented with 1% L-glutamine and 1% penicillin/streptomycin. Antigens were tested in duplicate with either 200,000 or 250,000 PBMC added to each well of the ELISpot plate. TRAP peptides were 20 amino acids in length, overlapping by 10 amino acids (NeoBioLab), assayed in six pools of seven to ten peptides at 10 μg/mL. Plates were counted using an AID automated ELISpot counter (AID Diagnostika GmbH, algorithm C), using identical settings for all plates, and counts were adjusted only to remove artifacts. Responses were averaged across duplicate wells, responses in unstimulated (negative control) wells were subtracted from each individual pool, then responses to individual pools were summed for each strain of the TRAP antigen. Responses to the negative control were always <142 SFC/10^6^ PBMC, with a median of 18 SFC/10^6^ PBMC. Pools were considered positive if the response was greater than the median plus 2 SDs (59 SFC/10^6^) of all negative control wells after subtraction of the autologous background. The lower limit of detection for the assay was 28 SFC for ME-TRAP. Staphylococcal enterotoxin B (0.02 μg/mL) and phytohemmagglutinin-L (10 μg/mL) were used as a positive control, whereby responses of >800 SFC/10^6^ passed quality control (QC). Reagents and methods were standardized between the two trial sites. Lymphocyte counts per milliliter of blood were taken directly from the hematology breakdown or calculated by multiplying the number of white blood cells per milliliter of blood by the lymphocyte differential percentage. These data were routinely obtained at post-vaccination time points as part of the safety assessments. This value was then multiplied by the number of SFC per million PBMC to produce the number of SFC per milliliter of blood.

### Flow Cytometry

PBMC were frozen in FBS containing 10% DMSO and stored in the vapor phase of liquid nitrogen. Of the 30 PBMC samples available in group 4, we selected samples where the ELISpot on fresh PBMC at day gave a response over 250 SFC per million PBMC, as responses lower than this would not be detectable by flow cytometry.

Thawing was performed rapidly in a water bath and cells were rested for 2 hr with benzonase at 25 U/10^6^ PBMC (Novagen) before stimulation overnight at 37°C with 5% CO_2_, either with Staphylococcal enterotoxin B, a pool of 56 peptides at 2 μg/mL spanning the entire length of the TRAP protein from the T9/96 strain of *P. falciparum* (Neopeptide) or an unstimulated control. Brefeldin A (BD Biosciences) 1 μg/mL and monensin (eBioscience) 1 μg/mL were added after 2 hr into the incubation and left to incubate for a further 16 hr. Cells were then washed in fluorescence-activated cell sorting (FACS) buffer (PBS containing 0.1% bovine serum albumin [BSA] and 0.01% sodium azide and stained for viability with LIVE/DEAD aqua amine reactive dye (Life Technologies) for 20 min at room temperature in the dark. Cells were then washed in FACS buffer and permeabilized for 20 min with Cytofix/Cytoperm (BD Biosciences) then washed in 1:10 permeabilization buffer (BD Biosciences). A cocktail of antibodies for surface and intracellular staining was added and incubated for 30 min at room temperature in the dark. The antibody cocktail is described in Table S2. Cells were again washed in permeabilization buffer and resuspended in PBS containing 1% paraformaldehyde, prior to acquisition on a BD LSR II on the day of staining. Compensation control beads (OneComp Beads, eBioscience, ArC Amine Reactive Beads, Invitrogen) were stained according to the manufacturer’s instructions using the same concentration of antibody used for cells to for compensation between parameters, and unstained cells were used to adjust forward and side scatter photo-multiplier tube voltages.

### Analysis of Flow Cytometry Data

At least 29,000 live CD3^+^ cells were analyzed per sample. Data were prepared and analyzed using FlowJo v9.6.2 (Treestar) with a hierarchical gating strategy. A sample gating strategy is shown in Figure S2. Responses to peptide were determined after subtraction of the response in the unstimulated control for each sample. Samples where a response to the positive control of >1% cytokine positive CD4^+^ or CD8^+^ T cells after subtraction of the unstimulated control could not be detected were excluded from the analysis. All samples had a minimum of 31,000 CD4^+^ or 8,000 CD8^+^ T cells in the parent population, thus the lower limit of detection for the assay was 0.0042% for CD4^+^ T cells and 0.022% for CD8^+^ T cells. A response was classified as positive if the response to peptide was greater than the medium control for the corresponding sample. Analysis of polyfunctionality was not undertaken due to low event numbers acquired from small pediatric samples.

### Anti-vector Neutralizing Antibody Assay

One day prior to performing the assay, GripTite 293 cells (Invitrogen) were seeded in 96-well plates (3 × 10^4^ cells/well). Heat inactivated test samples were diluted 4-fold from 1:9 to 1:2,304 in 10% FBS in DMEM and incubated 1:1 with ChAd63 expressing the secreted alkaline phosphatase gene (8 × 10^7^ vp/mL) for 1 hr at 37°C. Serum and virus were then added to 293 cells in a volume of 200 μL in duplicate for 1 hr, after which sample and virus were aspirated and replaced with fresh 10% FB DMEM. A virus-only control was included. After 22–26 hr at 37°C, 50 μL of medium was assayed for SEAP activity using a Phospha-Light TROPIX phosphatase assay (Applied Biosystems) in black assay plates, and luminescence was measured after 45 min on a Thermo-Fisher Varioskan Flash Luminometer. Anti-vector neutralization titers were defined as the dilution of serum showing 50% reduction in SEAP activity, based on observed % inhibition values relative to SEAP activity from virus alone. For trial A, anti-vector antibodies were measured in serum and for trial B plasma was used, however, we have determined that these sample types are equivalent for this assay.

### TRAP-Specific Total IgG ELISA

Standardized ELISAs for TRAP-specific antibodies were conducted as previously described^35^. Briefly, a reference standard of pooled anti-TRAP antibody positive serum was serially diluted to produce a standard curve, which was included on all plates. The standard sample was assigned a value in arbitrary ELISA units (EUs). The standard curve was then used to convert absorbance values of individual test sera (diluted to fall within the linear range of the curve) into EUs. A “seropositive cut-off” value was calculated using the mean plus three standard deviations of the EU values of 42 serum samples from unvaccinated UK volunteers. For the total IgG standardized ELISA, this cut-off value was 88 EUs.

### TRAP-Specific Isotype ELISA

Isotype ELISAs were conducted as previously described^53^ except plates were coated with 0.5 μg/mL of TRAP antigen in carbonate-bicarbonate coating buffer and left overnight at 4°C. Briefly, sera were diluted 1:100 in 0.2% BSA in PBS/Tween-20 (dilution buffer) and added in duplicate to each of six 96-well plates. After 2 hr, plates were washed and one of six secondary antibodies was added to each plate at 1:1,000 in dilution buffer, 50 μL per well. Secondary antibodies used were: biotin-conjugated mouse anti-human IgG1 Fc (clone HP6070) (Life Technologies); biotin-conjugated mouse anti-human IgG2 Fc (clone HP6002) (Life Technologies); biotin-conjugated mouse anti-human IgG3 (clone HP6050) (Sigma); biotin-conjugated mouse anti-human IgG4 (clone HP6025) (Sigma); alkaline phosphatase-conjugated goat polyclonal anti-human IgA a-chain (Sigma); and biotin-conjugated goat polyclonal anti-human IgM u-chain (Sigma). After 1 hr, plates were washed and 50 uL of ExtrAvidin alkaline phosphatase (Sigma) diluted 1:5,000 in dilution buffer was added to all plates (except IgA, to which only dilution buffer was added). After 30 min, all plates were washed and development buffer was added as for total IgG ELISA. Blank wells and internal development controls were included on each plate.

A “seropositive cut-off” value was calculated for each isotype or subclass using the mean plus 3 SDs of the EU values of 42 serum samples from unvaccinated UK volunteers. Cut-off values were 0.147 (IgG1), 0.166 (IgG2), 0.2002 (IgG3), 0.151 (IgG4), 0.362 (IgM), and 0.374 (IgA).

### TRAP-Specific IgG Avidity ELISA

IgG antibody avidity was assessed by sodium thiocyanate (NaSCN)-displacement ELISA. The assays were conducted as for total IgG ELISAs except that sera were individually diluted in dilution buffer to a level calculated to reach an OD405 of 1.0 (using total IgG EUs) and plated at 50 μL/well in 16 wells of a 96-well plate. Plates were incubated for 2 hr at room temperature (RT) before chaotropic agent NaSCN was added in duplicate at increasing concentrations down the plate (0–7 Molar [M]). Plates were incubated for 15 min at RT before washing, incubation with secondary antibody and development as for the total IgG assay. The concentration of NaSCN required to reduce the OD405 to 50% of that without NaSCN ( = IC_50_) was used as a measure of avidity. This was calculated from the intercept of the curve for each sample with the line of 50% reduction of the OD405 in the NaSCN-free well for each sample.

IgG1 and IgG3 avidity was assessed using the same protocol, except that two dilutions were made for each sample to levels calculated to reach an OD405 of 1.0 (using the OD values from the IgG1 and IgG3 ELISAs) and added to two plates, one of which was incubated with anti-IgG1 and the other with anti-IgG3. Secondary antibodies used were the same as used for the Isotype ELISAs.

### Statistical Methods

Group data display geometric mean or median. Matched pairs analyses were conducted where time points within a group were compared and excludes volunteers with missing data at any time point. UK and Gambian adult data were combined for several ELISpot and ELISA analyses due to no statistically significant differences between the two populations. A Kruskal-Wallis test was used to compare ELISpot and ELISA data in children against the adult control group, with Dunn’s multiple comparisons post-test. Kruskal-Wallis tests for comparisons between all groups were also used, with Dunn’s multiple comparisons post-test. For statistical analyses, an alpha-level of 0.05 was considered significant and all p values are two-tailed. All analyses were performed in GraphPad Prism, Mac version 6. (GraphPad Software).

## Author Contributions

Study Design, M.O.A., A.B.T., J.B.Y., S.H.H., N.A.A., C.J.A.D., K.L.F., B.K., S.B.S., K.B., A.V.S.H., I.N., and K.J.E.; Project Management and Regulatory Affairs, R.R., N.K.V., O.L., A.M.L., and E.B.I.; Provided Clinical Care to Participants, M.O.A., A.B.T., J.B.Y., and U.J.A.; Immunological Assay Design, C.M.B., G.B., N.J.E., K.L.F., A.V.S.H., I.N., and K.J.E.; Performed Experiments, C.M.B., A.D., G.B., G.S.S., Y.J.J., O.O., N.J.E., C.T., N.O., M.O., J.N.-J., A.D., I.N., K.J.E.; Data Analysis, C.M.B., G.B., N.J.E., I.N., and K.J.E.; Data Interpretation, C.M.B., G.B., A.V.S.H., I.N., and K.J.E.; Preparation of Figures, C.M.B., G.B., and K.J.E.; Manuscript Preparation, M.O.A., C.M.B., G.B., A.V.S.H., and K.J.E.

## Conflicts of Interest

The following authors have declared that no conflicts of interest exist: C.M.B., A.D., G.B., G.S.S., Y.J.J., O.O., N.J.E., C.T., N.O., M.O., J.N.-J., A.D., M.A.O., A.B.T., J.B.Y., J.U.A., S.H.H., N.A.A., R.R., C.J.A.D., R.C., N.K.V., O.L., A.M.L. K.L.F., B.K., E.B.I., S.B.S., K.B., I.N., and K.J.E. A.V.S.H. is a named inventor on patent applications on malaria vectored vaccines and immunization regimens. Authors from ReiThera are employees of and/or shareholders in ReiThera, which is developing vectored vaccines for malaria and other diseases.

## Supplemental Information

Document S1. Figures S1–S2 and Tables S1–S2

Document S2. Article plus Supplemental Information
