# Supplementary material for: Viral Vector Malaria Vaccines Induce High-Level T Cell and Antibody Responses in West African Children and Infants
Source: Mol Ther. 2017 Feb 22;25(2):547–59. doi: 10.1016/j.ymthe.2016.11.003 (PMC5368405; doi:10.1016/j.ymthe.2016.11.003)
Supplement: Document S1. Figures S1–S2 and Tables S1–S2 [file mmc1.pdf]

YMTHE, Volume *25*

Supplemental Information

Viral Vector Malaria Vaccines Induce High-Level T Cell and Antibody Responses in West African Children and Infants

Carly M. Bliss, Abdoulie Drammeh, Georgina Bowyer, Guillaume S. Sanou, Ya Jankey Jagne, Oumarou Ouedraogo, Nick J. Edwards, Casimir Tarama, Nicolas Ouedraogo, Mireille Ouedraogo, Jainaba Njie-Jobe, Amidou Diarra, Muhammed O. Afolabi, Alfred B. Tiono, Jean Baptiste Yaro, Uche J. Adetifa, Susanne H. Hodgson, Nicholas A. Anagnostou, Rachel Roberts, Christopher J.A. Duncan, Riccardo Cortese, Nicola K. Viebig, Odile Leroy, Alison M. Lawrie, Katie L. Flanagan, Beate Kampmann, Egeruan B. Imoukhuede, Sodiomon B. Sirima, Kalifa Bojang, Adrian V.S. Hill, Issa Nébié, and Katie J. Ewer

Supplemental material

a

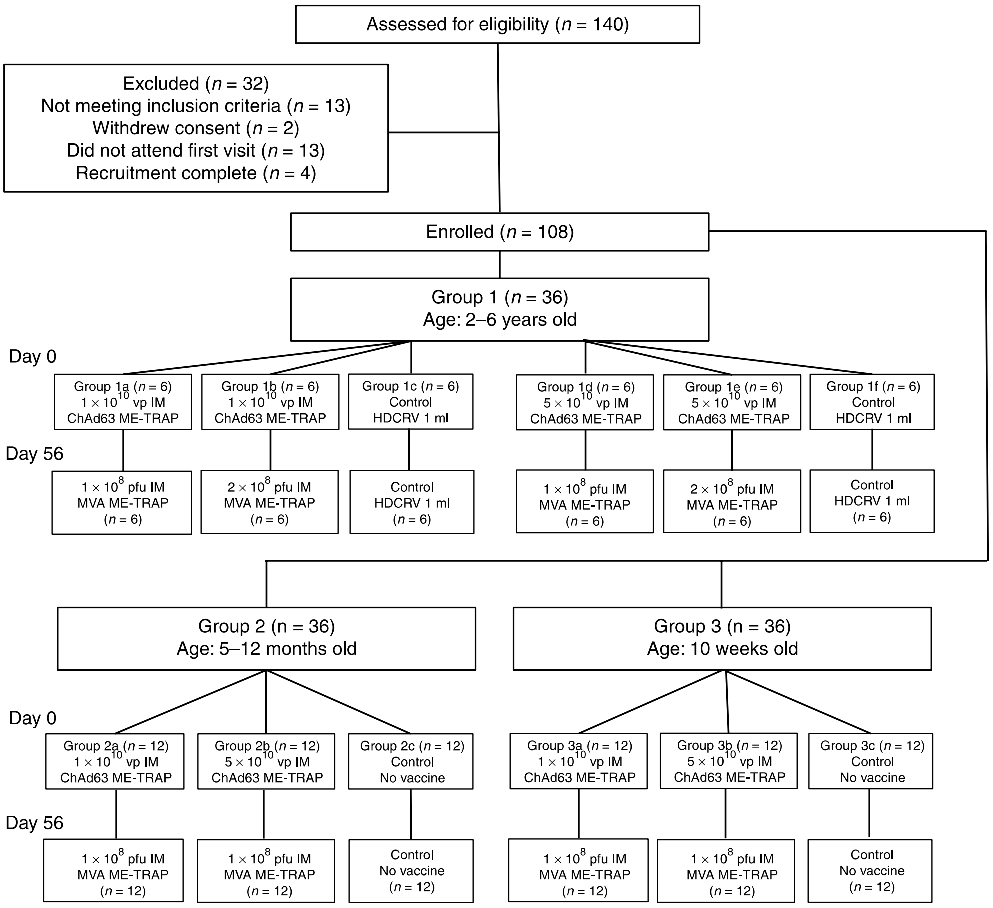

Figure 1a Clinical trial design for groups 1,2 and 3 in the Gambia.

b

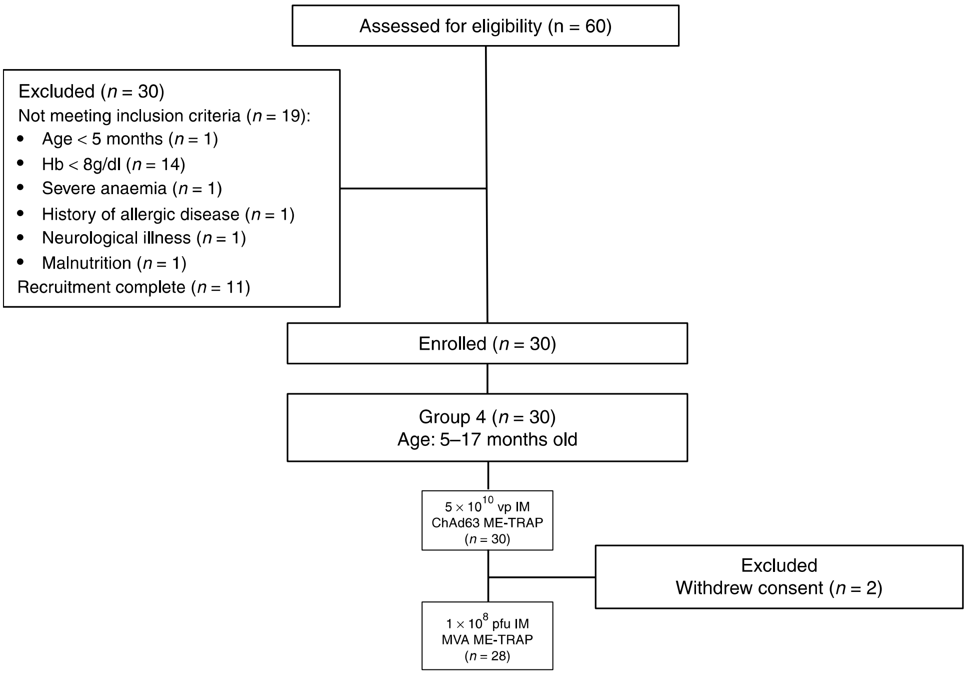

Figure 1b. Clinical trial design for Group 4 in Burkina Faso.

Supplementary Figure 1. Flow of study design and volunteer recruitment to trials in the Gambia (a) and Burkina Faso (b). All immunizations were administered intramuscularly with sequential vaccines administered into the deltoid of alternating arms. The parents of two enrolled volunteers in group 4 withdrew consent between vaccinations and consent was also withdrawn for one child in each of group 1a and 1b after vaccination before follow-up was completed; all other volunteers completed study visits as scheduled. Abbreviations: ChAd63, simian adenovirus serotype63; ME-TRAP, multiple epitope–thrombospondin-related adhesion protein; MVA, modified vaccinia virus Ankara; pfu, plaque-forming units; vp, viral particles; HDCRV, human diploid cell rabies vaccine.

Supplementary Figure 2. Sample gating strategy for flow cytometry.

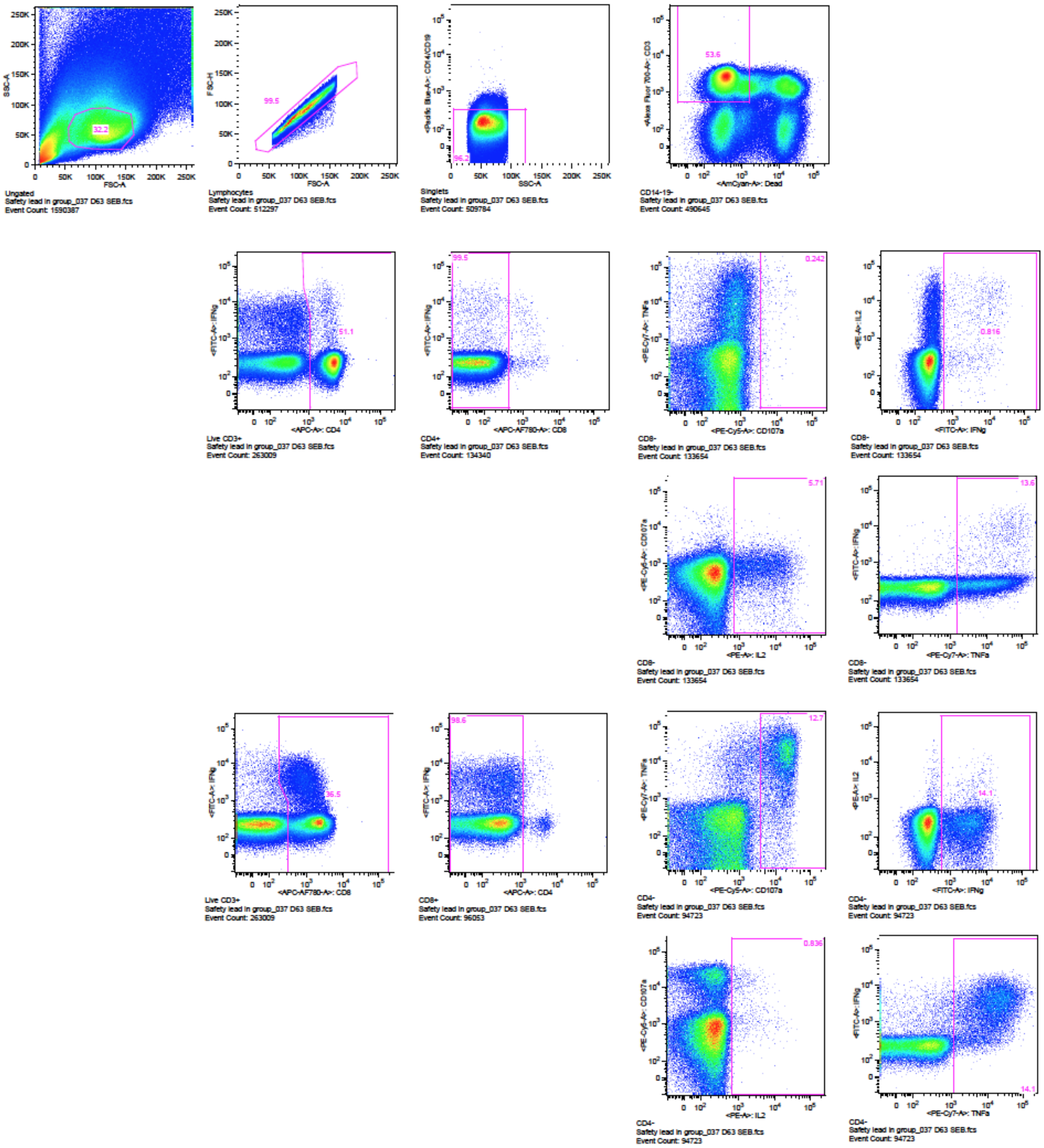

Supplementary Table S1. Baseline demographics of enrolled trial participants

							Group						
**Site**						The Gambia							Burkina Faso
**Age group**			2-6 years					5-12 months			10 weeks		5-17 months
**Study group**	1a	1b	1c	1d	1e	1f	2a	2b	2c	3a	3b	3c	4
**N**	6	6	6	6	6	6	12	12	12	12	12	12	30
**Median age, (IQR)**			44 months (36-51)					8 months (5.5-8.5)			10 weeks		44 weeks (36-61)
**Gender ratio (M/F)**			0.64 (23/13)					0.61 (22/14)			0.69 (25/11)		0.5 (15/15)

Supplementary Table S2. Antibodies used in flow cytometry.

	**Antibody**	**Fluorochrome /Dye**	**Clone**	**Supplier**	**Product code**	**Final Dilution**
		LIVE/DEAD	Aqua amine reactive dye	Invitrogen	L34955	1:200
	CD4	Allophycocyanin	RPA-T4	eBioscience	17-0049-73	1:50
	CD14	eFluor 450	61D3	eBioscience	48-0149	1:100
	CD19	eFluor 450	HIB19	eBioscience	48-0199	1:100
	CD3	Alexa Fluor 700	OKT3	eBioscience	56-0037-42	1:100
	CD8	APC-eFluor780	RPA-T8	eBioscience	47-0088-42	1:20
	IFNγ	FITC	4S.B3	eBioscience	11-7319-82	1:200
	IL-2	PE	MQ1-17H12	eBioscience	12-7029-82	1:100
	TNFα	PE Cy7	MAb11	BD Biosciences	25-7349-82	1:1000
	CD107a	PE-Cy5	eBioH4A3	eBioscience	15-1079-42	1:500
